# Development and Characterization of CASSAVA Starch-Based Biodegradable Films Reinforced with Kaolin and Andiroba Oil

**DOI:** 10.1021/acsomega.5c08831

**Published:** 2025-12-31

**Authors:** Mauricio Castro da Costa Filho, Jennypher Cristinne Souza Carneiro da Costa, Davi do Socorro Barros Brasil, Marlice Cruz Martelli

**Affiliations:** Faculdade de Engenharia Química, Instituto de Tecnologia, 37871 Universidade Federal do Pará, Rua Augusto Corrêa 01, 66075-110 Belém, Brasil

## Abstract

The effects of excessive consumption of nonrenewable packaging are detrimental to the environment and human health. In this context, the development of new biodegradable packaging incorporating active ingredients is a technological imperative; however, improving the mechanical and thermal properties of these materials remains a challenge. Therefore, the objective of this study was to develop and characterize biodegradable films based on cornstarch and cassava starch, incorporating andiroba oil and inorganic kaolin reinforcement, focusing on improving their physical, structural, barrier, and bioactive (antioxidant/antimicrobial) properties. The starchy materials presented morphologies consistent with those found in literature. The oil had an acidity index above that permitted by ANVISA, but while it was suitable for incorporation into the films, the other characteristics met the quality standards for vegetable oils. The kaolin presented particle size, crystalline structure, and morphology consistent with the literature. Biofilms were then developed using glycerol as a plasticizer and distilled water as a solvent, promoting the gelatinization process of the starch matrix and inorganic reinforcement of the kaolin. A factorial design was performed to evaluate the influence of starch mass, plasticizer mass, and kaolin mass on the thickness, water solubility, and water vapor permeability of the films, as well as to analyze their mechanical properties and biodegradability. It was demonstrated that the starch matrix mass and plasticizer mass were statistically significant for the behavior of the response variables studied. Furthermore, the inorganic reinforcement contributed significantly to improving the mechanical properties and biodegradability of active films. Therefore, the films obtained have potential for application in the development of biodegradable packaging that ensures higher quality packaged food.

## Graphical abstract

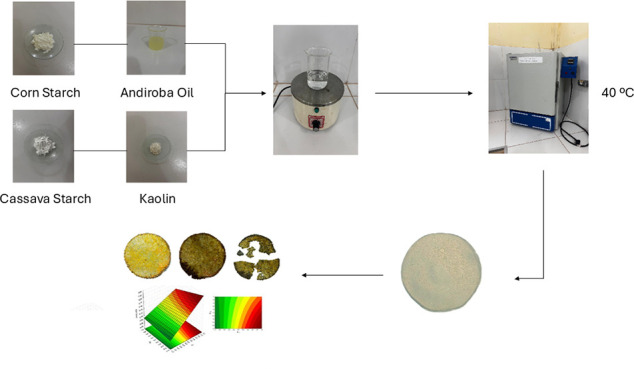

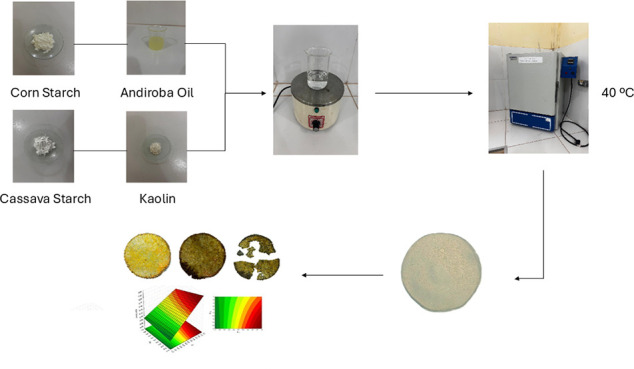

## 1 Introduction

Plastic is a polymer present in most products on the global market. Since its discovery, its use has been questioned. While it has promoted unparalleled technological advancement, its effects are extremely harmful to the environment and human health.^1^

Its use in the manufacture of disposable packaging, bags and wrappers promotes the unbridled and persistent accumulation of solid waste, which is highly biologically resistant,^2^ Furthermore, plastic is a strong pollutant of water bodies, soil and air.

In this sense, the discovery and research of new polymeric materials that are sustainable and biodegradable is essential, aiming to minimize waste without affecting product efficiency. Therefore, research into biopolymers from renewable sources is a very attractive alternative.^3^ Corn starch and cassava starch stand out, as they have great potential for the development of biodegradable films.^4^

Furthermore, the incorporation of active compounds that have antioxidant and antimicrobial properties, especially for casings intended for coating food.^5^ In this scenario, andiroba oil stands out as a possible antioxidant, antibacterial, antiseptic, emollient and insecticide agent, being widely used in the food and cosmetics industry.^6^

Still from this perspective, the need to reinforce these biodegradable polymers is highlighted, to ensure the improvement of their mechanical and thermal properties.^7^ Thus, kaolin stands out as a possible inorganic reinforcement for biodegradable films, as its three-dimensional crystalline arrangement positively impacts the mechanical and barrier properties of the films.^8^

In this context, this work aims to develop and perform physicochemical characterizations of biodegradable films based on corn starch and cassava starch, with incorporation of andiroba oil and inorganic kaolin reinforcement, focusing on improving their physical, structural, barrier and bioactive (antioxidant/antimicrobial) properties.

## 2 Methodology

Corn and cassava starch powder were purchased from a local supermarket chain in the metropolitan region of Belém, Pará, and used without any prior treatment to prepare the biodegradable active films. Analytical-grade glycerin was used as a plasticizing agent and distilled water as a solvent. The andiroba seeds were sourced from the municipality of Mãe do Rio, Pará (2°02′33.3″S, 47°33′22.2″W). The andiroba seed oil, to be incorporated into the biofilms, was extracted and characterized by the research group at the Synthesis Laboratory (LASIN) of the School of Chemical Engineering at the Federal University of Pará (UFPA). The kaolin used as structural reinforcement was donated by a mining company in the Amazon region.

### 2.1 Characterization of Raw Materials

The raw materials used were pretreated and characterized by laser particle size analysis, X-ray diffractometry (XRD), and scanning electron microscopy (SEM). The initial treatment consisted of washing the starchy material with distilled water. After 24 h, the supernatant was discarded, and the decanted mass was transferred to an oven (De Leo, model A3AFDI300) for drying at 40 °C for 24 h. The dried material was then sieved through 65 mesh sieves. The kaolin used was previously dried oven (De Leo, model A3AFDI300) at 105 °C to remove moisture. Kaolin particle size analysis was performed on a Litesizer DIF 500 particle size analyzer with ultrasonic liquid dispersion, using distilled water as the solvent. Kaolin diffractometry was performed on a PANalytical EMPYREAN diffractometer operating at 60 kV, with a 4 kW generator and PIXcel detector. Scanning electron microscopy analysis of cornstarch, cassava starch, and kaolin was performed on a ZEISS Sigma-VP scanning electron microscope equipped with a secondary electron and backscattered electron detector.

Andiroba oil was extracted by hot pressing at 60 °C in a hydraulic press weighing 10 to 12 tons, using 1 kg of oven-dried andiroba seeds (De Leo, model A3AFDI300) at 60 °C for 97 h. The pressed crude oil was characterized physicochemically, in triplicate, using nuclear magnetic resonance (NMR) analysis to determine its acidity, iodine, saponification, and peroxide indices, relative density, pH, and dynamic viscosity.

### 2.2 Production of Biodegradable Films and Factorial Design

To prepare the biodegradable films, a factorial experiment of the type 2^k^, with one replicate and three central points. The analyses were performed in triplicate, using as input variables the mass of the starch matrix, the mass of kaolin, and the mass of glycerol, both masses in grams. The design had as response variables the film thickness (mm), their solubility in water (%), and water vapor permeability (g·mm/kPa·m^2^·h). Table 1 presents the values used at each level of the variables studied in the factorial design.

**Table 1:** Values Used at Each Level of the Variables in the Factorial Design^a^

input variables	units	coded variables	–1	0	+1
starch matrix mass	g	SM	2.00	4.00	6.00
kaolin mass	g	KM	0.50	0.75	1.00
plasticizer mass	g	PM	0.40	0.80	1.20

a Source: Authors (2025).

The films were produced by gelatinizing the raw materials in 200 mL Erlenmeyer flasks containing 100 mL of distilled water and pouring the film-forming solution into circular molds for solvent dehydration in an oven at 40 °C.^9^

### 2.3 Characterization of Biodegradable Films

Film thickness (TK) measured using a MARBERG outside micrometer, with precision in the range of 0–25 mm, with ten measurements at different points of the biofilms.^10^ Water solubility analysis was performed using square specimens with side measuring 20 mm, previously dried in an oven, immersed in 30 mL of distilled water at room temperature for 24 h.^11^ The solubility of the films was calculated through eq 1.

1 $$\mathrm{SOL}=\frac{m_{0}-m_{f}}{m_{0}}\cdot 100$$

Where: SOL—solubility of films in water (%), *m*_0_—initial mass of test specimens (g) and *m*_f_—final mass of test specimens (g).

For the analysis of the permeability of films to water vapor, cells of permeability containing silica gel were sealed with films and kept in an environment with strictly controlled temperature and humidity for 24 h.^12^ The quantification of permeability was performed using eq 2.

2 $$\mathrm{WVP}=\frac{\left(\Delta W\cdot l\right)}{\left(t\cdot A\cdot \Delta P\right)}$$

Where: WVP is the water vapor permeability (g·mm/kPA·dia·m^2^), Δ*W* is the silica gel weight gain (g), *l* is the film thickness (mm), *t* is the analysis time (day), *A* is the biofilm surface area (m^2^) and Δ*P* is the vapor pressure difference across the film (kPa).

The films were characterized according to their mechanical properties, evaluating the maximum tensile strength (TS), elongation at break (EAB), and Young’s modulus (YM) in a universal texturometer at a speed of 20 mm/min, using triplicate specimens with dimensions of 80 × 20 mm^2^, conditioned in a desiccator for 24 h prior to analysis, and a separation space between the grips of 50 mm.^13^ To evaluate the results and the influence of kaolin particles as reinforcement, the results were compared with those of a control specimen containing all the constituents of the formulation except kaolin. In the biodegradability assessment, test specimens were buried and photographed periodically over a period of 3 weeks, allowing for the qualitative assessment of the biological degradation of the biodegradable film.^14^

## 3 Results and Discussion

### 3.1 Analysis and Characterization of Raw Materials

#### 3.1.1 Laser Diffraction (LD) Particle Size of Kaolin

Table 2 presents the results obtained in the diffraction particle size analysis. laser of the kaolin coating particles used in the work.

**Table 2:** Results of Laser Diffraction Particle Size Analysis of Kaolin^a^

*D*_10_	*D*_50_	*D*_90_	*D*_average_
0.823 μm	3.510 μm	7.970 μm	4.050 μm

a Source: Authors (2025).

From the analysis of the results, it was possible to verify that the kaolin sample analyzed presented a homogeneous granulometric distribution, with an average size of 4.05 μm particles. This granulometric range is a consequence of the micromorphological characteristics of the clay mineral particles, forming vermicular aggregates.^15,16^ The wide range of variations in particle size distribution observed in the results in Table 2 can be explained by the fact that LD analysis uses a dispersing agent that can increase the size of the analyzed particles, potentially leading to measurement errors. This is exacerbated when the analyzed particle has high polydispersity, and complementary techniques such as atomic force microscopy (AFM) are recommended.^17^

#### 3.1.2 X-ray Diffractometry (XRD)

The diffractogram in Figure 1 shows the diffraction pattern of the covering kaolin used in this research.

**Figure 1:**
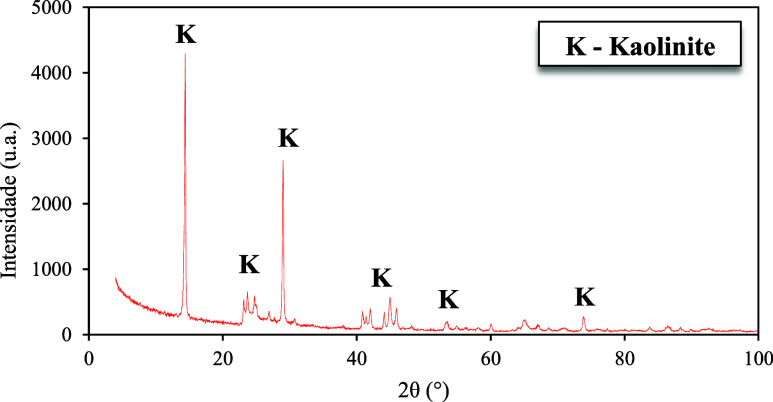
Diffractogram of kaolin. Source: Authors (2025).

The results indicate that the kaolin sample used presented as its majority composition kaolinite, with peaks located at 14.38° 2θ e 28.99° 2θ, results similar those obtained in the literature.^15–19^

#### 3.1.3 Scanning Electron Microscopy (SEM)

The morphology of corn starch, cassava starch and kaolin coating used in this work are presented, respectively, in Figure 2, 3, and 4. The corn starch granules had a smooth surface and a polyhedral or elliptical shape, without cracks or pores.^20,21^ The cassava starch granules were spherical or oval in shape, with smooth surfaces and slightly truncated ends, likely due to the mechanical extraction process. Furthermore, the birefringence pattern in the shape of a well-centered Maltese cross was preserved, indicating the preservation of their semicrystalline structure.^22,23^

**Figure 2:**
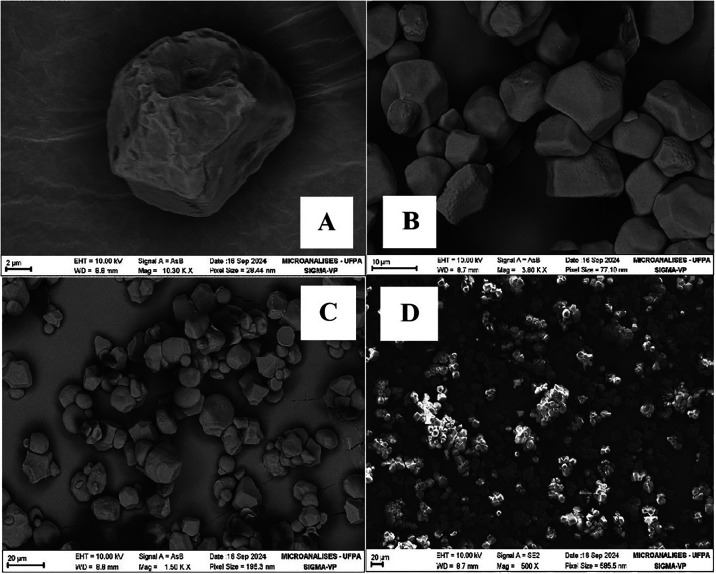
Micrographs of corn starch granules. (A) 10,300×; (B) 3800×; (C) 1500×; (D) 500×. Source: Authors (2025).

**Figure 3:**
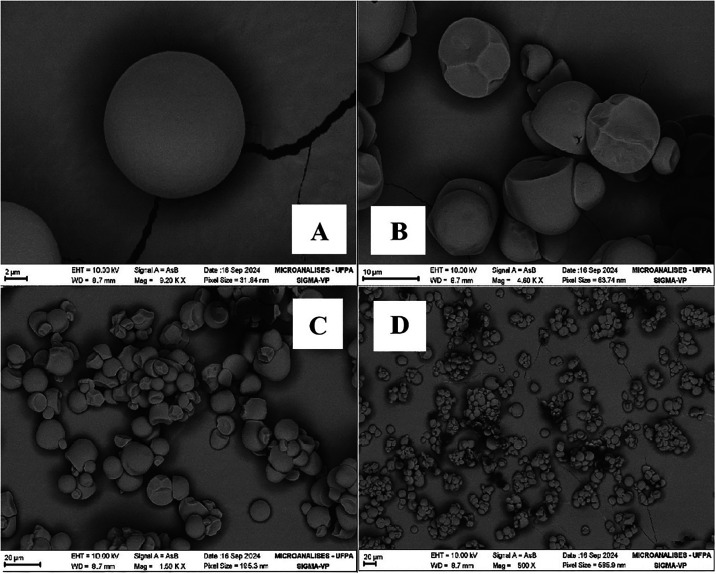
Micrographs of cassava starch granules. (A) 9200×; (B) 4600×; (C) 1500×; (D) 500×. Source: Authors (2025).

**Figure 4:**
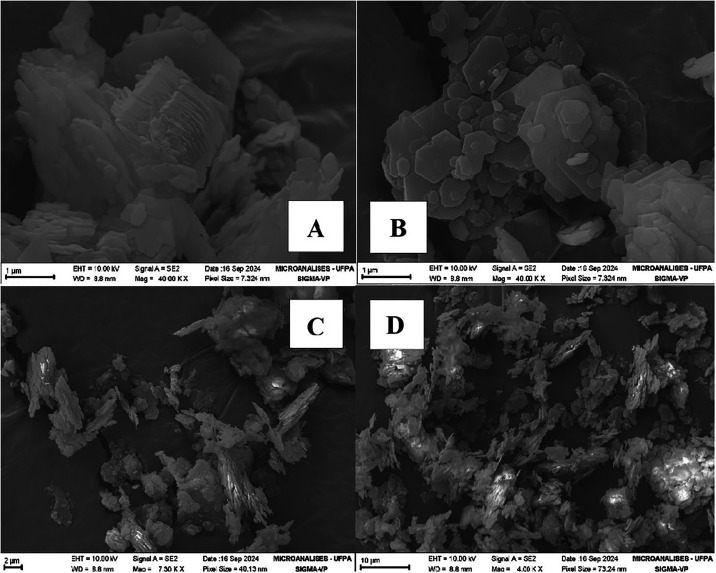
Micrographs of the covering kaolin. (A) 40,000×; (B) 40,000×; (C) 7300×; (D) 500×. Source: Authors (2025).

The kaolin particles were shaped like plates, formed due to the pseudohexagonal lamellar structure characteristic of the crystals of this clay mineral, with turbostratic arrangement of stacked crystallites and morphology.^15,16,24,25^

### 3.2 Analysis of Vegetable Oil

The andiroba oil used in the work presented a slightly yellowish coloration, with a clear appearance and free of visible impurities. The oil did not present visible signs of rancidity, with a characteristic almond odor.

#### 3.2.1 Nuclear Magnetic Resonance (NMR)

Nuclear magnetic resonance (NMR) identifies primary and secondary metabolites in a complex mixture. The H^1^ spectral profile of the oil allowed visualization of the chemical shift of the hydrogens in the triacylglycerol and identification of the protons present, as shown in Figure 5. The presence of doublets between δ_H_ 4.132 and 5.341 ppm was observed, attributed to the methylene protons of glycerol.^26,27^

**Figure 5:**
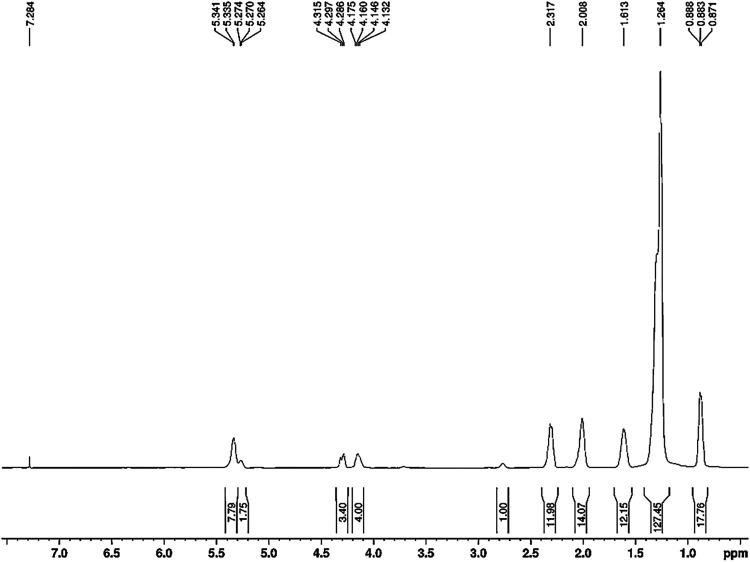
Chemical shift profile of andiroba oil by ^1^H NMR. Source: Authors (2025).

Furthermore, some signals in the ^1^H NMR spectrum could be attributed to chemical features of the structure of some fatty acids. This was the case of the chemical shift at δ_H_ 0.871 ppm, which indicated the presence of terminal methyl hydrogens of stearic acid, while the chemical shift at δ_H_ 0.888 ppm was attributed to palmitic and oleic acids.

These assignments can be confirmed by the chemical signals between δ_H_ 1.5 and 2.5 ppm, characteristic of the presence of β-methylene hydrogens of the carbonyl carbon, allylic methylene hydrogens, and methylene hydrogens adjacent to the carbonyl group present in stearic, palmitic, and oleic acids.^28^

#### 3.2.2 Physicochemical Characterization of Andiroba Oil

Table 3 presents the results of the physical-chemical characterization of the oil. The andiroba oil used had an acidity index above the limit stipulated by Anvisa^29^ for fixed oils of vegetable origin, suggesting the onset of a fermentation process that promotes enzymatic action related to the hydrolysis of triglycerides and the associated oxidative rancidity due to high humidity or light exposure during storage. High acidity index values indicate the low quality of the oil analyzed, suggesting the presence of other compounds such as citric or lactic acid because of the fermentation process, directly affecting the pH and organoleptic properties of the sample.^30^ Therefore, a low acidity index is essential in the development of casings to ensure the stability of food properties and consumer safety.

**Table 3:** Physicochemical Characterization of Andiroba Oil^a^

AI (mg KOH/g)	9.9150 ± 0.0919
II (g I^2–^/100 g)	58.1914
SI (mg KOH/g)	184.6556
PI (meq/kg)	1.7356 ± 0.2782
relative density	0.9173 ± 0.0011
pH	5.8 ± 0.1
dynamic viscosity (Pa·s)	0.1156 ± 0.0033

a Source: Authors (2025).

However, the other indexes were below the stipulated limits, enabling their use in incorporation into biodegradable films. The values of relative density, pH, and dynamic viscosity were consistent with results found in the literature for vegetable oils.^31,32^

### 3.3 Characterization of Biodegradable Films

#### 3.3.1 Factorial Design of Biodegradable Films

Table 4 shows the results obtained in carrying out experimental planning for the biodegradable films.

**Table 4:** Results of the Factorial Design of the Biodegradable Films Reinforced with Kaolin^a^

experiment	SM	KM	PM	TK (mm)	SOL (%)	WVP (g·mm/kPa·m^2^·h)
1	–1	–1	–1	0.060	22.68	1.4815
2	+1	–1	–1	0.209	15.41	3.7650
3	–1	+1	–1	0.075	16.78	1.8073
4	+1	+1	–1	0.147	14.04	5.5482
5	–1	–1	+1	0.105	30.45	2.2722
6	+1	–1	+1	0.223	18.77	4.3821
7	–1	+1	+1	0.109	37.35	2.1405
8	+1	+1	+1	0.182	18.15	4.7321
9	0	0	0	0.152	23.97	2.7408
10	0	0	0	0.133	22.53	4.9345
11	0	0	0	0.162	19.56	3.1817

a Source: Authors (2025).

In the casting method, controlling film thickness is essential to assess the distribution of constituents in the product. Thus, the films produced presented a homogeneous surface, free of cracks, easy removal, and without pores or bubbles. Figure 6 shows the influence of the factorial design variables on the thickness of cornstarch and cassava starch films. Film thickness ranged from 0.060 to 0.223 mm, a result in agreement with other studies that investigated the incorporation of reinforcing particles or oils in starch-based films.^33,34^ Film thickness was significantly influenced (*p* < 0.05, *R* ^2^ = 0.9838) by the mass of starch used in the formulation, with a tendency for film thickness to increase with increasing mass of starch used, as shown in Figure 6 (A,B).

**Figure 6:**
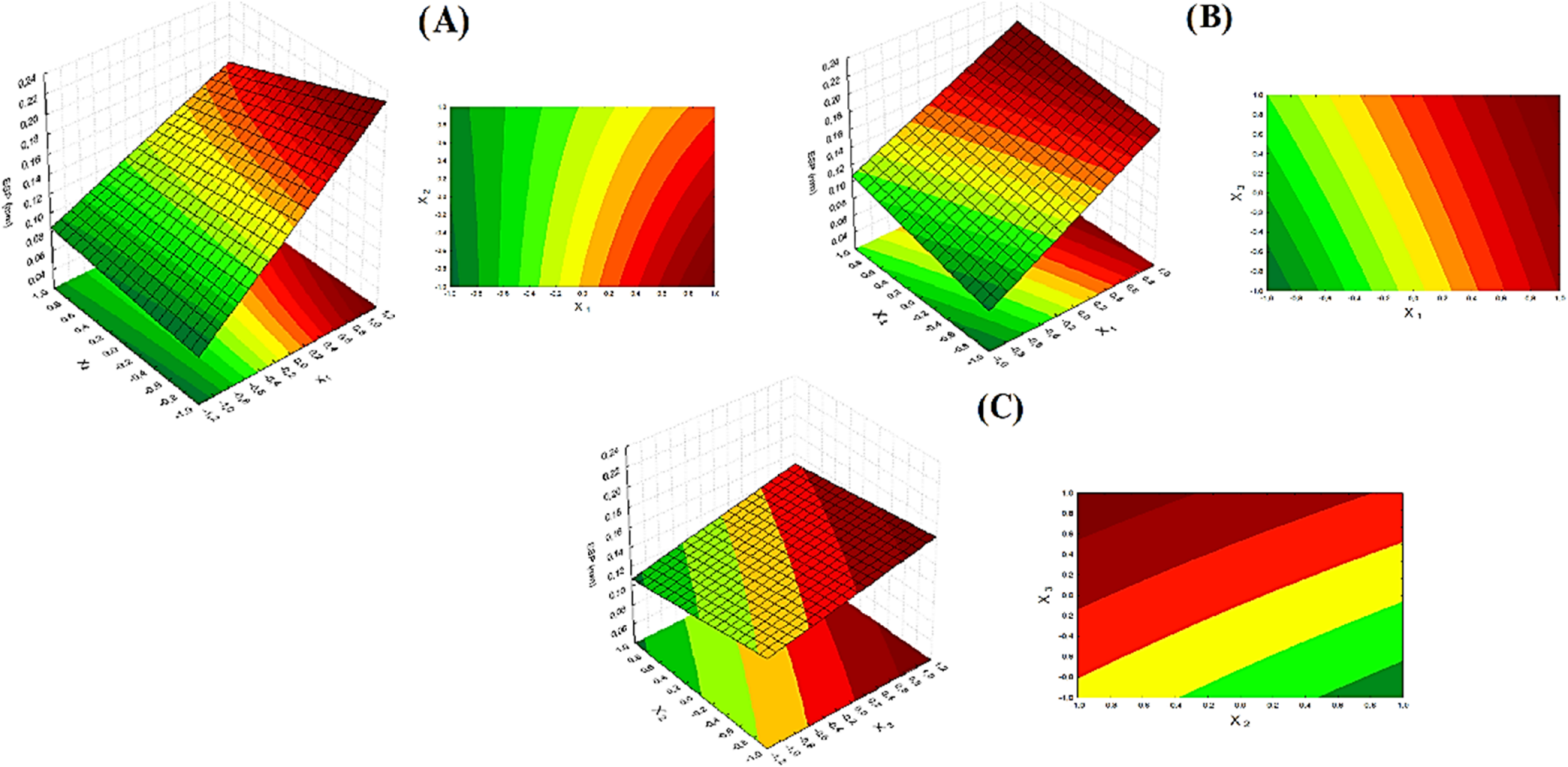
Response surface for thickness: (A) Starch mass × kaolin mass; (B) starch mass × plasticizer mass; (C) kaolin mass × plasticizer mass. Source: Authors (2025).

This can be explained by the fact that increasing starch mass in the formulation leads to an increase in total solids in the solution, promoting an increase in film thickness after dehydration.^35^ Furthermore, Figure 7 shows the distribution of residues from the thickness of biodegradable films. Analysis of the residues of the thickness of the biofilms indicated that their distribution occurs normally, with points very close to the line representing normality, low residual values and random pattern.

**Figure 7:**
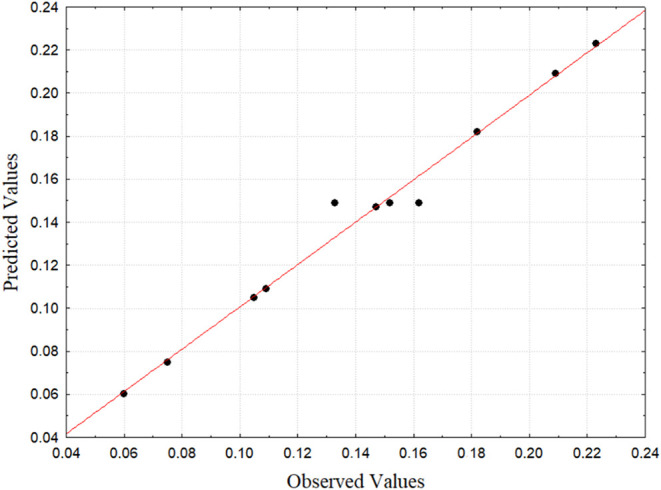
Distribution of residues for thickness.Source: Authors (2025).

Water solubility is a fundamental property for characterizing packaging, evaluating its behavior in environments exposed to this solvent. The films produced presented solubility ranging from 14.04 to 37.35%, values consistent with studies evaluating this property in starch films using glycerol as a plasticizing agent.^36,37^ This property was significantly influenced (*p* < 0.05, *R* ^2^ = 0.9788) by the starch and plasticizer masses in the formulation.

Film solubility tended to decrease as more starch was added to the formulation, as shown in Figure 8(A). This suggests that, as the starch mass increases, the starch polymer chains and their hydroxyl groups no longer interact as strongly with the solvent due to the saturation of the film-forming solution, affecting the diffusivity of water molecules in their structure, giving the product this hydrophobic characteristic.^38,39^ Furthermore, a strong interaction through hydrogen bonds occurs between the hydroxyl groups of the polymer matrix and the kaolin particles, with small particle size and high surface area, making the material more cohesive and reducing its interaction with the solvent.^8,16^ Regarding the plasticizer mass, water solubility increased as glycerol was added to the formulation, as observed in Figure 8 (B). This increase in solubility occurs because glycerol has hydrophilic properties,^40^ since due to its low molecular weight the plasticizer can widen the free spaces between the polymer chains, facilitating the entry of the solvent.^41^

**Figure 8:**
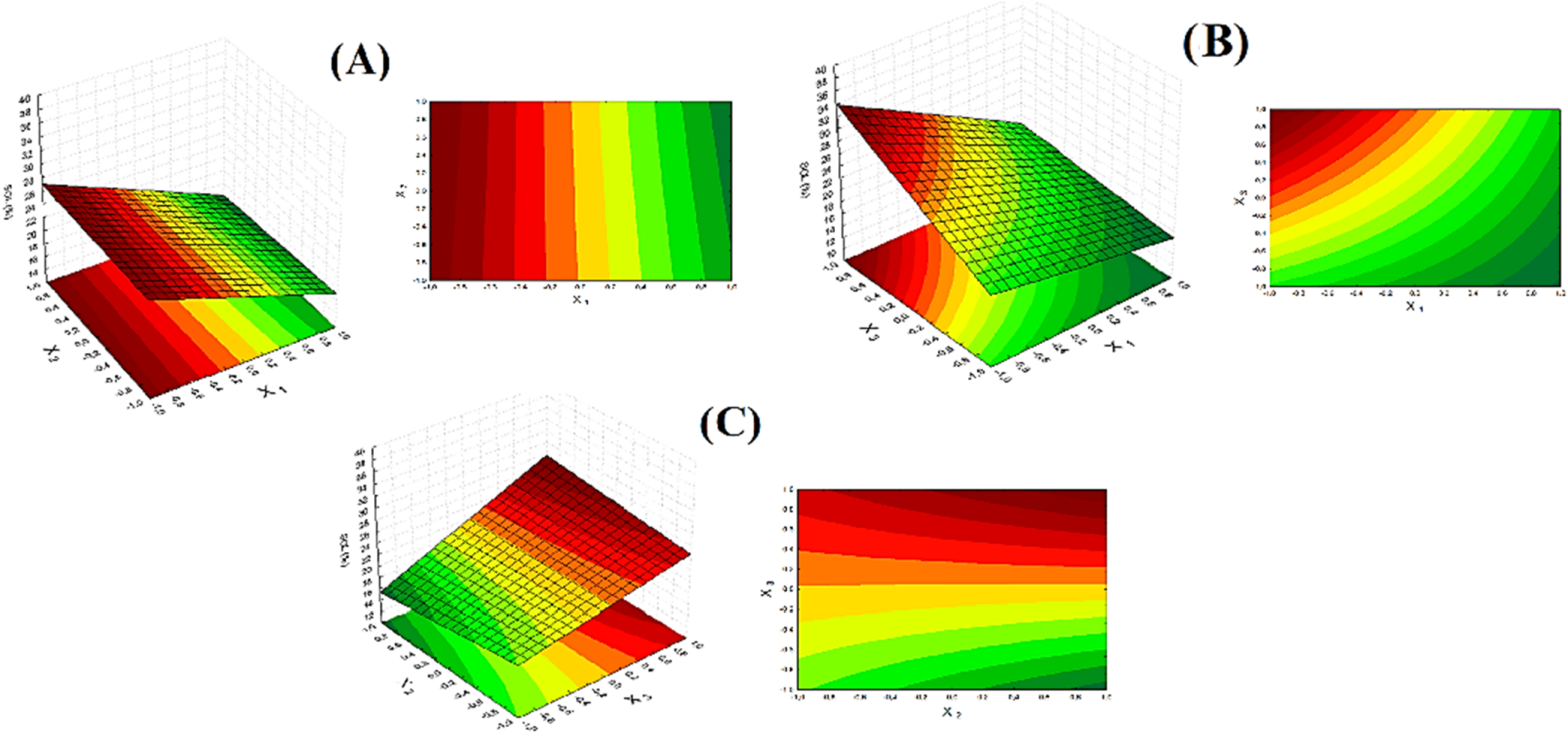
Response surface for water solubility: (A) Mass of starch × mass of kaolin; (B) mass of starch × mass of plasticizer; (C) mass of kaolin × mass of plasticizer. Source: Authors (2025).

**Table 5:** Results of the Values for the Tensile Test of the Films^a^,^b^

formulations	TS (MPa)	EAB (%)	YM (MPa)
control	24.7059 ± 0.0143	11.5271 ± 0.0544	214.3280 ± 71.9531
kaolin film	27.2373 ± 2.9551	7.6851 ± 1.8270	365.9963 ± 75.8857

a TS—Maximum rupture stress; EAB—elongation at rupture; YM—Young’s modulus.

b Source: Authors (2025).

Furthermore, Figure 9 shows the distribution of residues from the water solubility of biodegradable films. The analysis of the residues indicated, as in the thickness, that its distribution occurs normally, with points very close to the straight-line representing normality, low residual values and random pattern.

**Figure 9:**
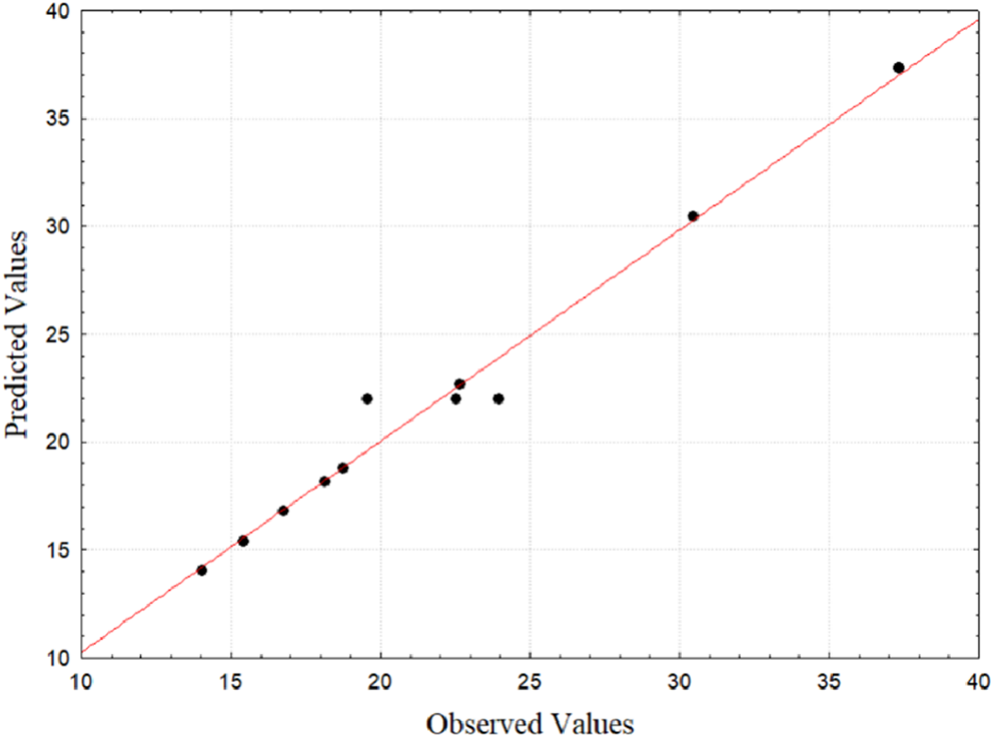
Residue distribution for solubility. Source: Authors (2025).

Water vapor permeability (WVP) is a property that allows us to study the behavior of films as a barrier to exposure to high-pressure and high-humidity environments. The WVP values for the developed films ranged from 1.4815 to 5.5482 g·mm/kPa·m^2^·h, consistent with other studies that investigated this property in cornstarch and cassava starch films.^42,43^ Statistical analysis revealed that WVP was not significantly influenced (*p* > 0.05, *R* ^2^ = 0.8611) by any of the variables studied in the factorial design, suggesting that variations in the starch, plasticizer, and kaolin mass in the formulation did not impact the film’s interaction with water vapor.

However, based on the analysis of the response surfaces in Figure 10, increasing the mass of starch and kaolin resulted in a slight increase in WVP. Some studies indicate that as film thickness increases, the distance that water vapor molecules must travel also increases, contributing to this impact on WVP.^44^ The reason this variation was not significantly relevant to the process may be attributed to the addition of andiroba oil to the formulation, which acts as a hydrophobic component and hinders the diffusion of water vapor molecules within the material structure.^45^

**Figure 10:**
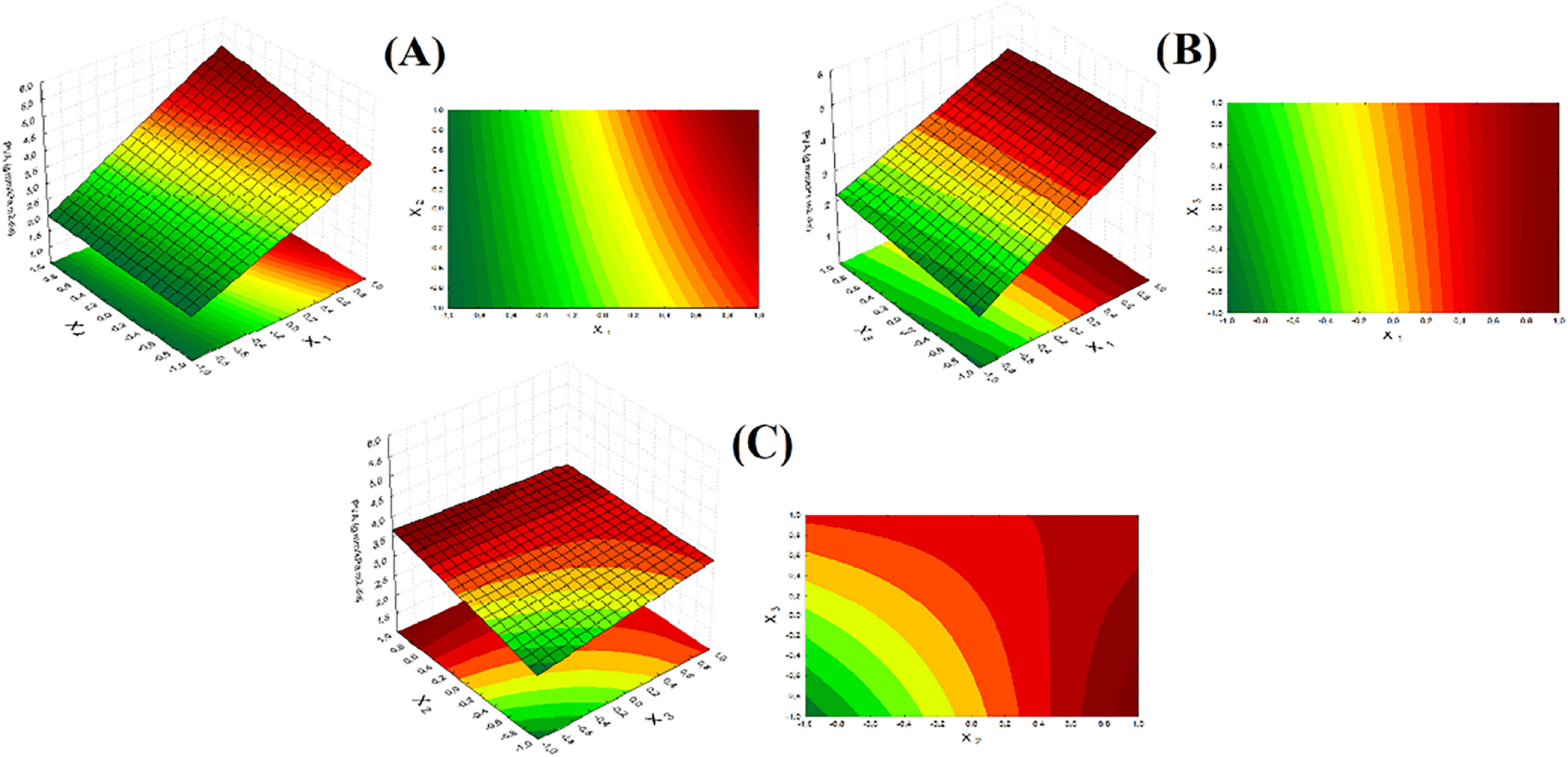
Response surface for WVP: (A) Mass of starch × mass of kaolin; (B) mass of starch × mass of plasticizer; (C) mass of kaolin × mass of plasticizer.

Figure 11 shows the distribution of residues from the water vapor permeability tests of the films. Analysis of the WVP residues indicated a normal distribution, with low residual values and a random pattern, but with points further from the normal line than the other response variables, as confirmed by the lower value of the model’s coefficient of determination.

**Figure 11:**
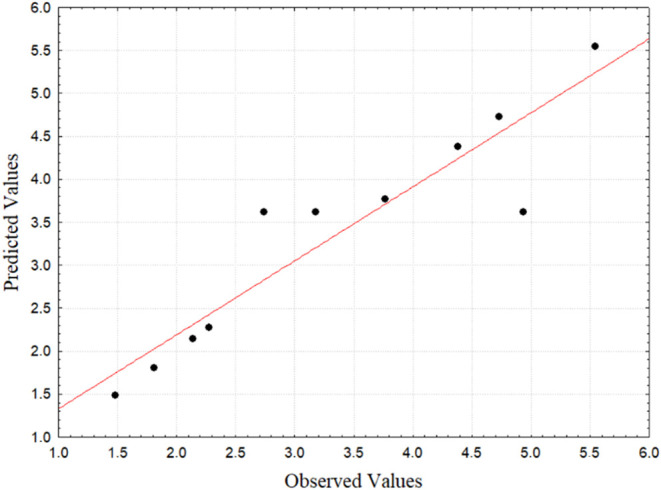
Waste distribution for WVP.Source: Authors (2025).

#### 3.3.2 Evaluation of the Mechanical Properties of Biofilms

Table 5 presents the average values obtained for the mechanical properties of the films produced. Studies that investigated the mechanical properties of starch films incorporated with andiroba oil^9^ and nitrosated starch incorporated with nitric oxide^46^ found results like those obtained in this study.

When compared with the control sample, the mechanical properties of films containing kaolin particles showed increased TS and YM, but reduced EAB. The incorporation of kaolin particles positively impacted the intermolecular interaction of the film structure, mainly due to the hydrogen bonds promoted in the polymer matrix, bringing the starch chains closer together and reducing the free volumes in its structure, explaining the increase in TS and, consequently, YM.^47^ The decrease in EAB is mainly due to this filling of the material structure, limiting chain mobility and film uniformity.^48^

Thus, the results obtained for the mechanical properties of the kaolin-reinforced films are much higher than those reported in the literature for starch films incorporated with andiroba oil,^9^ with TS values of 0.7293, EAB of 1.98% and YM of 36.80 MPa, indicating that the insertion of kaolin in the polymer structure was essential to ensure greater material resistance.

#### 3.3.3 Biodegradability

To investigate the biodegradability of the films produced, a qualitative assessment was performed, inspecting the disintegration of the film buried in the soil. Figure 12 shows the degradation of the film after different periods of burial. Visible changes were observed in the first week of analysis, with a significant color change. After the second week, the film began to fragment, due to the disruption of the interfacial interactions between the polymer structure and the kaolin particles, caused by soil moisture and biological activity.^49,50^

**Figure 12:**
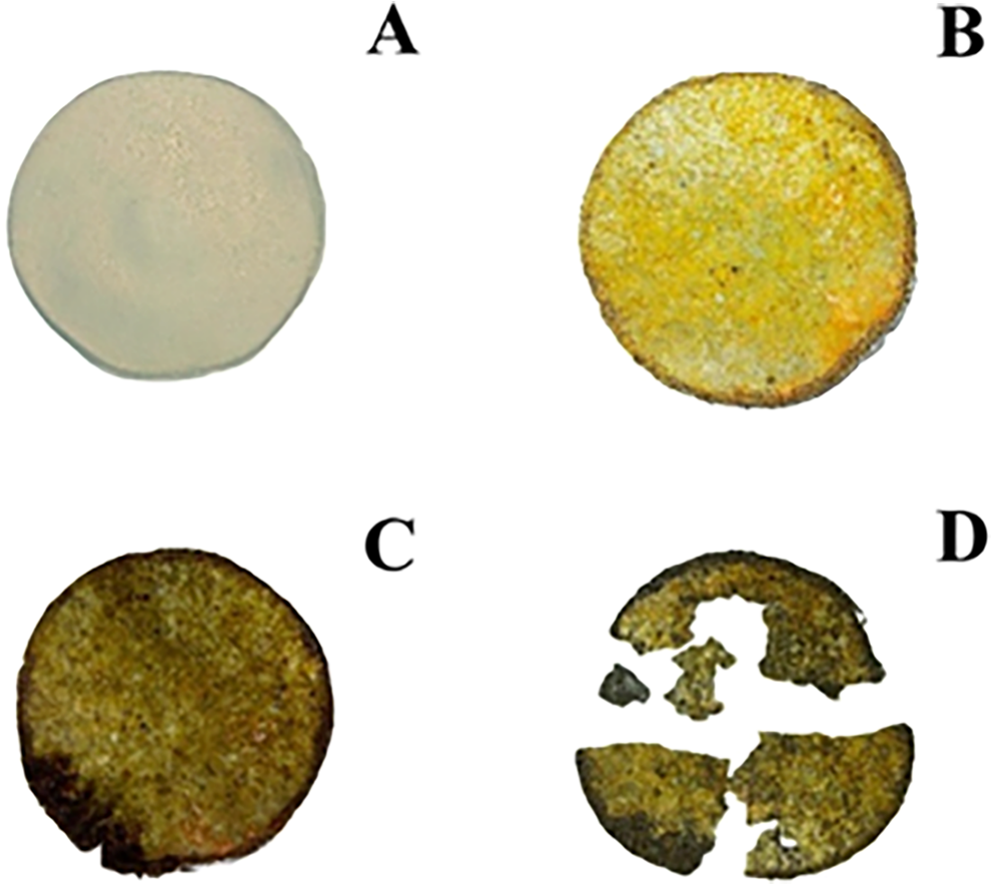
Appearance of the films before burial (A), first week of burial (B), second week of burial (C), and third week of burial (D). Source: Authors (2025).

Although the film degraded much of its initial mass after the third week of burial, this analysis does not provide quantitative evidence of its biodegradability. Future studies require the application of complementary techniques, such as monitoring the CO_2_ released during burial.^51^ However, the results obtained were essential in demonstrating that, even with kaolin reinforcement in their structure, the films maintained their biological degradation capacity, like other studies, proving superior to petroleum-derived polymers in terms of sustainability.^52^

## 4 Conclusions

In the work carried out, it was possible to demonstrate success in the development and characterization of corn starch and cassava starch biofilms, with incorporation of andiroba oil and inorganic kaolin reinforcement. It was found that the addition of andiroba oil andiroba to the biofilm, favored its properties, especially its biodegradability. In addition, the kaolin filler favored the mechanical properties and barrier of the film, without interfering with its elasticity and malleability. In short, it was concluded that the polymeric film produced in this work proved to be a possible alternative for replacing synthetic plastic packaging biodegradable, presenting active ingredients that can contribute to increasing shelf life of packaged foods. It is important to note that the application of films produced in food coating is an aspect that can be explored in future work.

## References

[1] de
Lima A. C., Frost V. C. A., Cardoso F. A. R. (2024). The Use of Starches
in the Production of Biodegradable Products: A Review with Temporal
Space. Rev. Gestão Soc. Ambientale.

[2] Rossini, L. A. ; François, A. L. ; Magalhães, B. S. G. ; Santos, T. R. D. Synthesis and characterization of biofilm based on pine nut starch Rev. Thêma et Sci. 2025 10.37136/ths.v15i1.2108.

[3] Melo S. N., Nascimento F. C. A., Silva N. M. M., da Silva Pessoa M. M., dos Santos Conceição G., Guimaraes S. C. N., Brasil D. S. B., de Arimateia Rodrigues
do Rego J. (2025). Biodegradable
films made from cassava starch: an analysis of sustainable innovations. Revista Aracê.

[4] de Castro, J. M. C. Use of Biofilms in Post-Harvest Conservation of Pineapple (Ananas comosus): A Literature Review, TCC (bachelor’s degree in Agronomy); Instituto Federal Goiano: Ceres, Goiás; 2025.

[5] da Silva, L. M. A. ; Alves, J. J. L. ; Fernandes, C. C. Obtaining and characterizing biodegradable films based on cornstarch incorporated with purple guava essential oil Revista Eletrônica Interdisciplinar 2025 10.29183/2238-8869.2024.v14i2.p178-193.

[6] Rufino, J. P. F. ; Araújo, B. S. ; Carneiro, S. B. ; Lima, E. S. ; Guimarães, C. C. ; Silva, J. L., Junior ; Chaves, F. A. L. ; Mendonça, M. A. F. ; Costa neto, P. Q. Quality of eggs convered with biofilms containing different levels of andiroba oil and stored at room temperature Braz. J. Poult. Sci. 2024 10.1590/1806-9061-2023-1887.

[7] Ramos, G. S. S., Junior Synthesis and characterization of biodegradable polymeric nanocomposites for application in the packaging sector. Dissertation (Master in Materials Science and Engineering), Universidade Federal do Pará (UFPA): Ananindeua, 2025.

[8] Jafarzadeh S., Alias A. K., Ariffin F., Mahmud S., Najafi A. (2016). Preparation
and characterization of bionanocomposite films reinforced with nano
kaolin. J. Food Sci. Tecnol..

[9] da
Costa filho M. C., Brasil D. S. B., Martelli M. C. (2024). Study of the variation
of andiroba oil concentration (Carapa guianenses Aublet.) on the properties
of cornstarch and cassava starch biofilm. Rev.
Observatório Econ. Latinoam..

[10] de Medeiros, A. S. Evaluation of Hydrophilicity in Cornstarch Films with the Addition of Beeswax. TCC (bachelor’s degree in Agricultural Engineering), Universidade Federal do Rio Grande do Norte (UFRN): Macaíba, 2025.

[11] Araújo, A. C. L. Synthesis of Ionic Liquids Based on Biomolecules and Their Application in the Formulation of Starch Films. TCC (bachelor’s degree in chemistry), Universidade Federal da Paraíba: Areia, 2018.

[12] American Society for Testing and Materials . Standard Test Method for Water Vapor Transmission of Materials; E96-16, 2016.

[13] American Society for Testing and Materials . Standard Test Method for Tensile Properties of Thin Plastic Sheeting; D882, 2012.

[14] de
Carvalho Silva M. T., Cunha P. C. D., Farias C. S. D., da Costa Machado M. T., da Rocha Ferreira E. H. (2023). Development
of a smart and biodegradable film from purple-fleshed sweet potato
for qualitative food evaluation. Rev. Obs. Econ.
Latinoam..

[15] Sousa B. B., Rego J. A. R., Brasil D. S. B., Martelli M. C. (2020). Síntese e
caracterização de zeólita tipo sodalita obtida
a partir de resíduo de caulim. Cerâmica.

[16] Mochiutti E., Sehwartza R. L. C., Lima J. P. O., Carvalho A. L. S., Neves R. F., Brasil D. S. B., Martelli M. C. (2020). Implementação do campo
de força Clayff no GROMACS: uma aplicação em
estrutura de caulinita. Quim. Nova.

[17] Kgabi D. P., Ambushe A. A. (2023). Characterization of South African Bentonite and Kaolin
Clays. Sustainability.

[18] Martelli M.
C., Neves R. F. (2012). Characterization
and Technological Applications for Kaolin Processing Wastes. Mater. Sci. Forum.

[19] Mañosa J., Rosa J. C., Silvello A., Maldonado-Alameda A., Chimenos J. (2023). Kaolinite structural modifications induced by mechanical
activation. Appl. Clay Sci..

[20] Davoudi Z., Azizi M. H., Barzegar M. (2022). Porous corn
starch obtained from
combined cold plasma and enzymatic hydrolysis: Microstructure and
physicochemical properties. Int. J. Biol. Macromol..

[21] Jiang X., Zheng F., Yu J., Lv P., Ban H., Liu H., Cai D., Xiu L., Liu J. (2025). Effects of static magnetic
field treatment on the digestive, structural and physicochemical characteristics
of germinated corn starch. Food Chem..

[22] Arroyo-Dagobeth E. D., Cadena-Chamorro E. M., Figueroa-Flórez J. A., Salcedo-Mendoza J. G., Serna-Fadul T. Y., Ortega-Quintana F. (2025). Synergistic heat-moisture and enzymatic
modification of starch blends: a case study on structuring cassava-based
gluten-free baked goods. Appl. Food Res..

[23] Silva R. S. O., Figueiredo H. M., Rodrigues A. M. C. (2025). Production
of Lactobacillus plantarum ATCC 8014 and microencapsulation using
cassava starch and Bactris Gasepaes Kunth palm starch. LWT.

[24] Yang Y., Jaber M., Michot L. J., Rigaud B., Walter P., Laporte L., Zhang K., Liu Q. (2023). Analysis of the microstructure
and morphology of disordered kaolinite based on the particle size
distribution. Appl. Clay Sci..

[25] Zaccaron, A. Synergistic Effect of High-Energy Grinding and Organic Intercalation on the Structural Evolution and Properties of a Kaolin. Thesis (Doctorate in Materials Engineering), Universidade do Extremo Sul Catarinense (UNESC): Criciúma, 2024.

[26] Di
Pietro M. E., Mannu A., Mele A. (2020). NMR determination of
free fatty acids in vegetable oils. Process.

[27] da
Silva N. M., Lopes I. C. S., Galué-Parra A. J., Ferreira I. M., de Sena C. B. C., da Silva E. O., de Matos
Macchi B., de Oliveira F. R., Nascimento J. L. M. (2024). Fatty
acid amides suppress proliferation via Cannabinoid receptors and promote
the apoptosis of C6 Glioma cells in association with Akt Signaling
Pathway inhibition. Pharmaceuticals.

[28] de
Sousa Monteiro I., Fonseca A. S. A., dos Santos C. R., de Carvalho J. P. S., da Silva S. W., Veiga V. F., Ribeiro R., Vieira I. J. C., Nogueira T. S. R., da Costa C. A. R. (2025). The development and
characterization of an Andiroba Oil-Based nanoemulsion (Carapa guianensis,
Aubl.): Insights into its physico-chemical features and in vitro potential
healing effects. Pharmaceutics.

[29] BRAZIL . Resolution RDC/ANVISA/MS No. 87, of March 15, 2021. Technicalregulation for vegetable oils, vegetable fats, and vegetable cream. Official Gazetteof the Federative Republic of Brazil. Brasília, DF, March 15, 2021. Section 1. 2021.

[30] Lozano-Garzón K., Orduz-Díaz L. L., Guerrero-Perilla C., Quintero-Mendoza W., Carrillo M. P., Cardona-Jaramillo J.
E. C. (2023). Comprehensive Characterization
of Oils and Fats of Six Species from the Colombian Amazon Region with
Industrial Potential. Biomolecules.

[31] de Lima, F. S. Use of Andiroba Oil (Carapa guianenses) for Soap Production in Combination with Aqueous Ash Extract. TCC (bachelor’s degree in chemistry); Universidade Federal do Pará (UFPA): Ananindeua, 2022.

[32] de Alencar, F. L. Andiroba Oil (Carapa guianenses Aublet.): from the botanical characteristics of the species to oil extraction. TCC (bachelor’s degree in Biological Sciences), Universidade Federal do Amazonas (UFAM): Tacoatiara, 2023.

[33] Lopes H. S.
M., Costa F. A. T., Mathias S. L., Sillard C. B., Dufresne A., Komatsu D., Menezes A. J. (2025). High-Elongation Starch Films by Hydroxypropylation
Reaction with Low Glycerol Content. ACS Omega.

[34] Raj J. S. A., Kasi M., Karuppiah P., Hirad A. H. (2024). Green Packaging
Solutions for Extending the Shelf Life of Fish Fillet: Development
and Evaluation of Cinnamon Essential Oil-Infused Cassava Starch and
Fish Gelatin Edible Films. ACS Omega.

[35] Teseme W. B., Habtegebreil S. A., Tolesa G. N. (2025). Study on the selected engineering
properties of anchote (*Coccinia abyssinia*) starch based biodegradable film for food packaging. Appl. Food Res..

[36] Silva V. D. M., Macedo M. C. C., Rodrigues C. G., Santos A. N., Loyola A. C. F., Fante C. A. (2020). Biodegradable edible
films of ripe banana peel and
starch enriched with extract of *Eriobotrya japonica* leaves. Food Biosci..

[37] Martins S. H. F., Pontes K. V., Filgueiras C. T., Nogueira G. F., Fialho R., Fakhouri F. M. (2025). Development and
characterization of starch films extracted
from avocado (*Persea americana mill*) seeds incorporated
with avocado (*Persea Gratissima*) essential
oil. Int. J. Biol. Macromol..

[38] Islam H. B. M. Z., Susan M. A. B. H., Imran A. B. (2020). Effects of Plasticizers
and Clays on the Physical, Chemical, Mechanical, Thermal, and Morphological
Properties of Potato Starch-Based Nanocomposite Films. ACS Omega.

[39] Chaves M. L. C., Jesus G. A. M., Castro M. C., Bruni A. R. S., Monteiro J. P., Santos O. O., Martins A. F., Bonafé E. G. (2025). Biodegradable
Pectin/Starch-Based Films Applied on Fresh Pears. ACS Omega.

[40] Asikkutlu A. G., Yildirim-Yalcin M. (2025). Optimization of mechanical and water
barrier properties
of avocado seed starch based film and its application as smart pH
indicator by adding blue butterfly pea flower extract. Food Chem.: X.

[41] Crema N. M., Castro L. E. N., Alves H. J., Ribeiro L. F. (2025). Impact of incorporating
chitosan into starch films: analysis of physicochemical properties
and degradability in soil. Food Chem. Adv..

[42] Lopes A. C., Klosowski A. B., Olivato J. B. (2025). Production and Characterization of
Biodegradable Wound Dressings Containing Silver-Loaded Zeolite Complexes. ACS Omega.

[43] Dmitrenko M., Pasquini D., Bernardo M. P., de Lima
Alves J. M., Kuzminova A., Dzhakashov I., Terentyev A., Dyachkov A., Joshy K. S., John M. J., Thomas S., Penko A. (2025). Bio-Composite Films from Carrageenan/Starch
Reinforced with Nanocellulose
for Active Edible Food Packaging: Development and Optimization. J. Renewable Mater..

[44] Torche A., Chouana T., Bensalem S., Khaled M., Rekbi F. M. L., Kelai E., Uzun S. A., Sarıcaoglu F. T., D’Elia M., Rastrelli L. (2025). Cassava Starch–Onion
Peel
Powder Biocomposite Films: Functional, Mechanical, and Barrier Properties
for Biodegradable Packaging. Polymers.

[45] Sartori T., Menegalli F. C. (2016). Development and characterization of unripe banana starch
films incorporated with solid lipid microparticles containing ascorbic
acid. Food Hydrocolloids.

[46] da
Silva J. F., Almeida E. A., Karoleski G. E., Koloshe E., Peron A. P., Job A. E., Leimann F. V., Shirai M. A., Gonzalez R. S. (2024). Synthesis of a Bioactive Nitric Oxide-Releasing
Polymer from S-Nitrosated Starch. ACS Omega.

[47] Othman S. H., Zaid N. S., Shapi’i R. A., Nordin N., Talib R. A., Tawakkal I. S. M. A. (2025). Starch biopolymer films containing carbon black nanoparticles:
Properties and active food packaging application. J. Sci.: Adv. Mater. Devices.

[48] Tanpichai S., Thongdonson K., Boonmahitthisud A. (2023). Enhancement of the mechanical properties
and water barrier properties of thermoplastic starch nanocomposite
films by chitin nanofibers: Biodegradable coating for extending banana
shelf life. J. Mater. Res. Technol..

[49] Dong M., Mastroianni G., Bilotti E., Zhang H., Papageorgiou D. G. (2024). Biodegradable
Starch-Based Nanocomposite Films with Exceptional Waterand Oxygen
Barrier Properties. ACS Sustainable Chem. Eng..

[50] Bouzidi S., Lévèque J. M., Magnin A., Molina-Boisseau S., Putaux J. L., Boufi S. (2024). Thermoplasticized
Bread Waste and
Poly­(butylene adipate-co terephthalate) Blends: A Sustainable Alternative
to Starch in Eco Friendly Packaging. ACS Sustainable
Chem. Eng..

[51] AMerican Society for Testing and Materials . Standard Test Method for Determining Aerobic Biodegradation of Plastic Materials in Soil; D-5988, 2012.

[52] Tavares A. V. W., Soares D. M. V. G., Pereira R. C. S., Pontes
Neto A. M. A., de Vilhena A. E. G., Nascimento S. C. C., Brasil D. S. B., Martelli M. C. (2024). Obtaining and characterizing biodegradable
films based on potato starch and glycerol using Box-Behnken design. Rev. Obs. Econ. Latinoam..

